# On Variation of Polyandry in a Bush-Cricket, *Metrioptera roeselii*, in Northern Europe

**DOI:** 10.1673/031.013.1601

**Published:** 2013-03-02

**Authors:** Peter Kaňuch, Berrit Kiehl, Matthew Low, Anna Cassel-Lundhagen

**Affiliations:** 1 Department of Ecology, Swedish University of Agricultural Sciences, Box 7044, 750 07 Uppsala, Sweden; 2 Institute of Forest Ecology, Slovak Academy of Sciences, L'. Štúra 2, 960 53 Zvolen, Slovakia

**Keywords:** body mass, copulation frequency, latitude, Orthoptera, seasonality

## Abstract

Patterns of polyandry in nuptial-gift-giving insects are often explained in terms of sexually antagonistic coevolution. However, the potential influence of environmental constraints and life-history traits on polyandry in these species is still largely unexplored. As an initial step in examining the role of these factors, this study measured the number of matings (spermatodoses per female) of female Roesel's bush-crickets, *Metrioptera roeselii* Hagenbach (Orthoptera: Tettigoniidae), along a latitudinal gradient in northern Europe (16 sites, 53.89–60.47° N). Females contained between 0 and 5 spermatodoses (mean ± SE: 1.7 ± 0.08; *N* = 114), with the degree of polyandry generally increasing at higher latitudes (approximately 0.12–0.3 matings per degree of latitude). As expected, female body size also had an influence on polyandry; the number of matings increased from small to moderately large individuals before declining. The field-based results suggested that there were potentially interesting interactions between environment, life-history traits, and patterns of polyandry in nuptial-gift-giving insect species, and these potentially interesting interactions are used to outline future research directions.

## Introduction

In nuptial-gift-giving insects, females greatly benefit from multiple matings (Arnqvist and Nilsson 2000; Gwynne 2001) but a large range in the degree of polyandry is exhibited between and within species (Bretman and Tregenza 2005; Vahed 2006). Inter-specific comparisons of polyandry and sexual physiology have been largely responsible for the current understanding of mating patterns and sexual conflict in such insects (Vahed 2006, 2007a). However, in order to understand the degree to which these mating patterns are shaped by life-history traits or environmental constraints, within-species between-population comparisons under different environmental conditions need to be considered. One of the few approaches in this direction has been to use latitudinal variation in polyandry to test how life-history traits interact with sexual conflict and the control of reproduction in the green-veined white butterfly (Välimäki and Kaitala 2006, 2010; Välimäki et al. 2006; also see Hodkinson 2005 for a review on the relationship between altitude and fecundity in insects). Unfortunately, there is a general paucity of published data on geographical differences in polyandry in insects (e.g., Välimäki and Kaitala 2006, 2010; Välimäki et al. 2006).

Bush-crickets from the family Tettigoniidae (Orthoptera) are nuptial-gift-giving insects whose reproductive behavior and its associated sexual conflict have been carefully studied (see Gwynne 2001; Vahed 2006, 2007b and references therein). Males stridulate to attract females for mating and then facilitate the transfer of their ejaculate by secreting a gelatinous nuptial gift (spermatophylax) for the female to eat (Vahed 1998; Gwynne 2001). These nuptial gifts are costly to produce (Vahed 2007b) but are used by the male to help overcome female resistance in accepting large ejaculates and may result in greater sperm transfer and higher offspring production (Ivy et al. 1999; Fedorka and Mosseau 2002). Females may resist large ejaculates because they have a fitness cost via their dose-dependent inhibition of female receptivity to remating (Gwynne 1986; Wedell 1993). Another important factor that influences mate choice, and consequently fecundity, is the relative body size of females (Gwynne 1984; Honěk 1993). Larger females mate more frequently because of their ability to outcompete smaller individuals (Brown 2008). In addition to sexual conflict, environmental conditions may influence patterns of polyandry through constraints on production of the spermatophylax and ejaculate, and by influencing the sex ratio of the population (Gwynne 1984; Gwynne 1990).

As a first step in considering environmental conditions on polyandry in insects, the degree of polyandry in female Roesel's bush-cricket, *Metrioptera roeselii* Hagenbach (Orthoptera: Tettigoniidae), along a latitudinal gradient in northern Europe was examined. The advantage of using bush-crickets for polyandry research is that the number of matings in field-collected females can be estimated directly by counting the number of spermatodoses within the females' spermathecae (Gwynne 2001; Vahed 2006). A spermatodose, spermatophore-like structure is formed after each copulation; it envelops the male ejaculate and remains within the spermatheca for the duration of the female's life (Vahed 2003).

Latitudinal decline in temperature is the main factor affecting species phenology in northern Europe (Rötzer and Chmielewski 2001). In the present study, local environmental conditions in the north (i.e., microclimate, season length) are less favorable for embryonic and nymphal development and time to sexual maturity when compared to the south (Ingrisch 1984; Ingrisch and Köhler 1988), with the species showing latitude-responsive traits related to body growth (Cassel-Lundhagen et al. 2011). Thus, the aim of this study was to examine relationships between polyandry and local environmental conditions (i.e., latitude) in *M. roeselii,* while accounting for other factors that can influence mating frequency (i.e., body size and time of season). From the direction of this relationship, explanatory factors to help guide future studies in this field are proposed.

## Materials and Methods

### Study species

*M. roeselii* is a widespread grassland orthopteran species in continental Europe, and is currently expanding rapidly towards the north of Fennoscandia. Adult body length ranges from 14–18 mm, and they usually appear as flightless short-winged morphs. In northern Europe, adult bush-crickets die at the beginning of each winter with the next generation reaching reproductive maturity in the following summer (July–August; Ingrisch and Köhler 1998). Genetically isolated populations of *M. roeselii* tend to have larger bodies at higher latitudes; however, no such latitude-body size relationship is observed in females within the species' continuous range (Cassel-Lundhagen et al. 2011). Univoltine and semivoltine life cycles may coincide in northern latitudes, where eggs laid early in the summer develop into adults within one year, while those laid in autumn need two seasons to complete their life cycle (Ingrisch 1984). The level of polyandry in this species is thought to be relatively low (mean 2.6, *N* = 6; Vahed 2006), but this is based on a very small sample size.

### Data collection

**Figure 1.**
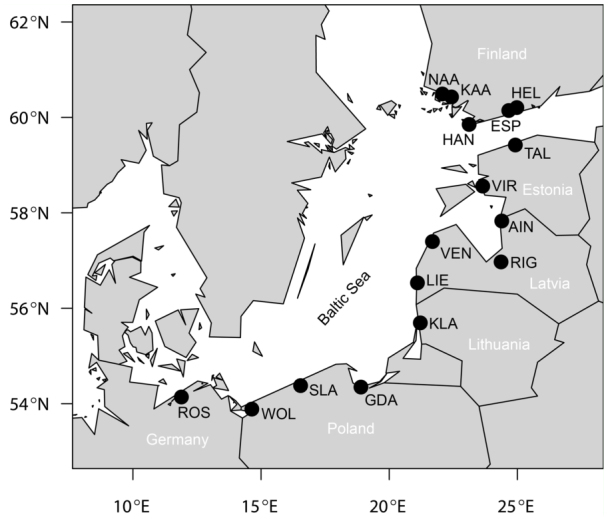
Sampling sites of *Metrioptera roeselii* females along the Baltic Sea coast in northern Europe. For full names and latitudes of the site abbreviations, see Table 1. High quality figures are available online.

In the present study, 114 reproductively-active adult female bush-crickets were collected from 16 sites along the eastern and southern Baltic Sea coast of Finland, Estonia, Latvia, Lithuania, Poland, and Germany (53.87– 60.47° N, 11.96–24.87° E; Figure 1, Table 1). These sites were chosen because: (1) they span seven degrees of latitude (Table 1), a range seemingly adequate for detecting climate-demographic relationships in insects (e.g., Edwards et al. 2009; Rhainds and Fagan 2010); (2) they are all subject to coastal climate, thus limiting confounding interactions between climatic conditions of inland versus coastal sites at different latitudes; and (3) all sites are within a region where the species is widespread and where high levels of gene flow have been detected, thus any phenotypic variation should be due to strong selection pressures (Cassel-Lundhagen et al. 2011) or plastic responses to local conditions.

**Table 1.**
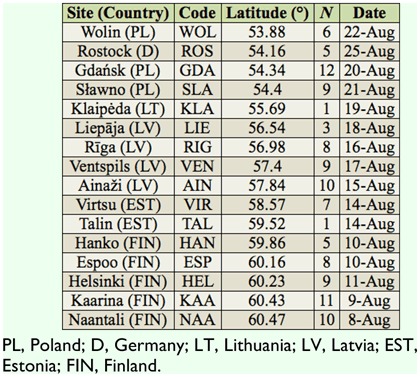
Sampling sites, sampling site abbreviation code (for Figure 1), sampling site latitude (in decimal degrees), site-specific sample sizes (N), and the date of collection for the female *Metrioptera roeselii* collected along the Baltic Sea coast in 2008 for this study.

**Table 2.**
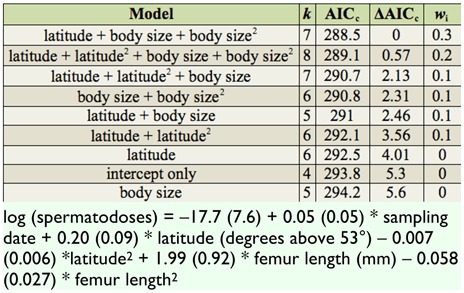
Candidate model set showing relative support for the effect of female body size (femur length) and latitude on the number of copulations (i.e., spermatodoses) in female *Metrioptera roeselii.* Models are ranked according to the Akaike information criterion corrected for sample size (aicc) and also show the number of parameters being estimated (*k*), the model ranking relative to the best model (Δaicc), and the relative strength of support for each model (*w*i)*.* All models included an overdispersion parameter, sampling date as a fixed effect and sample population as a random effect. The aic-weighted model-averaged parameter estimates (means with standard errors in parentheses) are given in the Table footnote.

**Figure 2.**
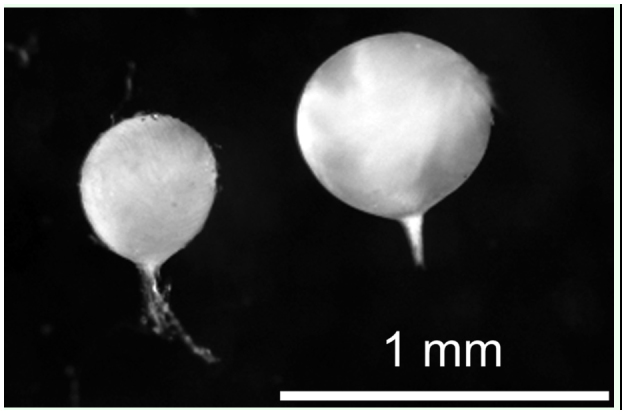
Spermatodoses dissected from the spermatheca of an adult *Metrioptera roeselii* female. Each spermatodose represents a separate successful insemination. High quality figures are available online.

All bush-crickets were caught using hand nets between the 8^th^ and 25^th^ of August 2008 (Table 1). The timing of capture allowed females several weeks to find males and copulate before being captured and preserved in 99% ethanol. To estimate the number of copulations each female had engaged in, the spermathecae from the females' abdomens were dissected (in Ringer's solution under a 16× binocular enhancer), and the number of spermatodoses within was counted. For this study, the generally accepted assumption was made that each spermatodose represents one copulation and vice versa (Gwynne 2001; Vahed 2006). In *M. roeselii,* spermatodose size ranges in diameter between 0.5 and 1 mm. It is white and onion shaped, consisting of a thin outer layer and gelatinous inner layer of sperm mass (Figure 2). To assess the body size of the females, the length of the hind femur was measured by the same person using a digital slide calliper (accuracy ± 0.03 mm) under a magnifying (3×) lamp.

### Data analysis

Generalized linear mixed models were used to examine the influence of latitude and body size (femur length) on the number of spermatodoses (polyandry) in female bush-crickets. All models included sampling date as a continuous fixed effect to account for possible age-related biases (because females at northern sites were sampled much earlier in their development than females in southern sites) and sample population as a random effect to account for repeated measures at the 16 sampling sites (all individuals at each site were collected on the same day). The models were fitted using a quasi-Poisson distribution to constrain estimates above zero, penalized quasi-likelihood estimation was used, and over-dispersion was corrected for by adjusting the distribution variance based on overdispersion estimates (0.36 ± 0.05). Because the two fixed factors (i.e., body size and latitude) had potential non-linear relationships with copulation frequency, both linear and quadratic terms were tested in the models. The nine candidate models were compared and ranked using the Akaike information criterion corrected for sample size with parameter estimates for model predictions derived from model averaging based on Akaike information criterion model weights (Burnham and Anderson 2002). These calculations were performed in MLwiN version 2.20 (Rasbash et al. 2009) and plots were created using R 2.11.1 (R Development Core Team 2008).

## Results

Females contained between 0 and 5 spermatodoses in their spermatheca (mean 1.7 ± 0.08 SE; 43.0% contained one, 34.2% two, 17.5% three, 0.9% four, and 0.9% five, while 3.5% contained none (*N* = 114)). There was a higher level of polyandry in the northern half of the study area (57–61° N; 61.4%, *N* = 70) when compared to the southern half (53–57° N; 40.9%, *N* = 44).

**Figure 3.**
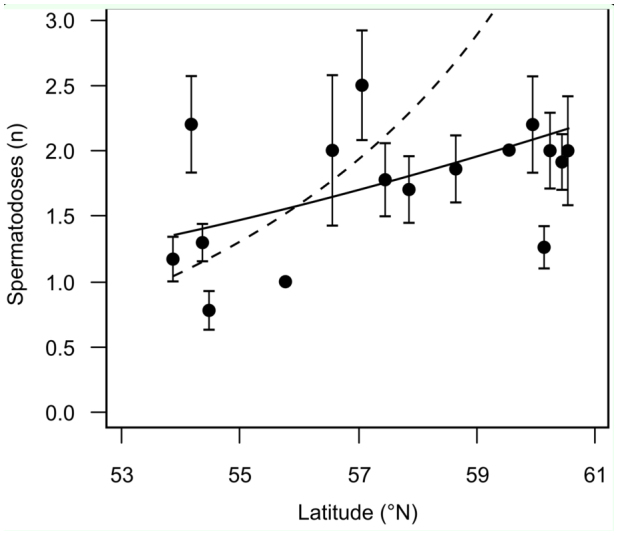
Number of spermatodoses per adult female (mean ± SE) at each sampling site relative to latitude (see Table 1 for sample sizes) with body size held constant. The prediction lines are derived from the highest-ranked model in Table 2. The solid line shows the relationship when sampling date was not corrected for; the dashed line is the model prediction when sampling date in this study was corrected for. High quality figures are available online.

Support was found for the number of spermatodoses per female to increase at higher latitudes (Table 2; Figure 3; Akaike information criterion relative-importance weight of latitude = 0.86); this relationship was largely linear (Figure 3), as the inclusion of a quadratic term did not improve model support (Table 2). The positive effect of latitude on matings was not an artifact of sampling date, because northern sites (where some individuals were still in nymphal stages) were sampled earlier than southern sites (Table 1); thus, northern bush-crickets were at an earlier stage in their mating season and should, all else being equal, have shown a trend towards having less matings rather than more. This effect could be demonstrated by comparing parameter estimates for latitude from the highest-ranked model in Table 2 (which included a sampling date correction) to the same model without sampling date being corrected for. Latitude was important in both models; however, when sampling date was not corrected for, the estimate for the increase in matings per degree of latitude was conservative (mean (± SE) estimates for sampling-date-corrected versus uncorrected: log (spermatodoses) = 0.19 (± 0.09) versus 0.07 (± 0.03) * degrees north of 53°; see Figure 3).

**Figure 4.**
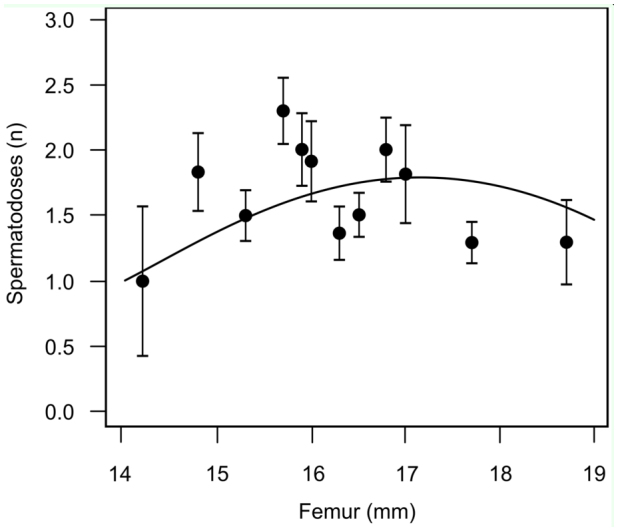
Number of spermatodoses per adult female (mean ± SE) relative to body size (femur length in mm) with the effects of latitude held constant. The raw data are clumped into 12 categories to aid visual representation. The prediction line is derived from the AIC-weighted model-averaged parameter estimates in Table 2. High quality figures are available online.

There was also support for a relationship between female body size and the number of spermatodoses per female (Table 2; Figure 4). The highest number of spermatodoses were found in crickets of intermediate size (∼15–17 mm; Figure 4), with there being stronger support for models that included a quadratic term in addition to a simple linear term (from highest-ranked model in Table 2; log (spermatodoses) = 2.96 ± 1.38 * femur length (mm) - 0.087 ± 0.04 * femur length^2^).

## Discussion

Female *M. roeselii* had higher levels of polyandry at higher latitudes, a pattern contrary to that seen in another nuptial-gift-giving insect, the green-veined white butterfly (Välimäki and Kaitala 2006, 2010; but see Devaux and Lachaise 1987). The pattern in our study could not be explained as an artifact of sampling date, because the bush-crickets were sampled from north to south; thus, those from northern latitudes were at an earlier stage of their mating season (some undeveloped nymphs still occurred at the time of collection in the north). Also, there was no evidence that this result was confounded by larger body sizes affecting the mating frequency of northern populations (cf. Gwynne 1984; Honěk 1993; Brown 2008). The populations lie within the northern boundary of the continuous distribution of this species in Europe, where latitude does not affect average body size in a positive way (Cassel-Lundhagen et al. 2011). If anything, the bush-crickets in this study were slightly smaller in the north (femur length (mm; mean estimate ± SE) = 17.1 (± 0.2) - 0.2 (± 0.05) * degrees north of 53°). Since there was no difference in mating frequency between the largest and smallest females, it confirms that the there was no competition pressure relative to body size. This suggests that the latitudinal relationship is real, and there are three nonexclusive factors that should be examined in future studies of this species and other insects where males use nuptial gifts to influence female remating rates.

The first is to consider the relationship between environmental conditions (latitude was used as a proxy for climatic conditions in this study) and male nuptial gift / ejaculate size (Välimäki and Kaitala 2010). The production of nuptial gift secretions is costly (Thornhill 1980; Vahed 2007b) and should therefore be related to resource availability, the time available for mating, and male longevity. In northerly environments, resources may be limited and/or the time available for reproduction highly restricted (see Hodkinson 2005 for similar relationships with altitude). In such situations, both males and females may change their behavior in order to maximize their respective fitness within the shortened mating season (Gwynne 1993; Gwynne et al. 1998). If males change their behavior by reducing the amount of material they transfer during mating, it would also result in lower levels of copulation-inhibiting substances being transferred to the female. This should allow her to re-mate more often (Gwynne 1986; Vahed 2006). However, if the limited resources lead to smaller size of the spermatophylax, then the fitness benefits gained from the additional resources can be reduced, causing the female to mate multiple times (Ivy et al. 1999; Fedorka and Mosseau 2002). It is also worth considering that larger nuptial gifts not only indicate larger amounts of inhibitory substances (Vahed 2006), but also prolonged attachment of the sperm-filled ampulla and greater sperm transfer (Sakaluk 1984; Wedell 1991). The amount of transferred sperm itself then has an impact on the refractory period of the female, as bigger spermatodoses fill the reproductive tract of the female and limit further copulations (Simmons and Gwynne 1991). However, since there are no data on nuptial gift sizes, this hypothesis needs to be confirmed when such data become available.

The second consideration is the relative strength of selection on the timing of mating and how it changes with latitude. In *M. roeselii,* southerly populations are univoltine (i.e., they complete one generation per year), while more northerly latitudes have an increasing proportion of semivoltinism (i.e., individuals that take an additional year to develop into adults; Ingrisch 1984). In areas where univoltine and semivoltine life histories coincide, there should be strong selection on individuals to start mating early in the season, as offspring of such early maturing individuals can complete their life cycle in one year rather than two (Stearns 1992). However early maturation can be energetically costly, and in areas where the semivoltine life strategy prevails, individuals may better be able to allocate resources towards copulation frequency (cf. Simmons et al. 1992).

The final factor is the relative fitness advantage for females living in an unpredictable environment (e.g., Goddard et al. 2005; Wilson 2009). In northern latitudes, prolonged embryonic development might increase the risk of mortality due to longer exposure to environmental impacts. From the female perspective, an increased frequency of copulations can then increase the chances for her to copulate with more genetically-compatible males and thus produce more viable offspring (Tregenza and Wedell 1998; Fedorka and Mosseau 2002; Bretman and Tregenza 2005; Bretman et al. 2009). For example, Fedorka and Mosseau (2002) found that females of the ground cricket, *Allonemobius socius,* had a more than twofold increase in hatching success and a more than 40% greater offspring survivorship in polyandrous females compared to monandrous ones. There is little empirical evidence regarding the influence of temperature on polyandry, but Wilson (2009) found that more copulations maintained individual reproductive success in colder environments in a *Syngnathus* fish (an organism that is Poikilothermic, like insects). Apart from the production of more genetically diverse offspring, this greater reproductive success could also work through a larger portion of extra nutrients gained from more than one spermatophylax (Vahed 1998; Gwynne 2001). This theory requires, however, that a more stressful environment causes males to produce less inhibitory nuptial gifts (as discussed above).

It should be noted that our study only offers a snapshot of the degree of polyandry. Although this study does not elucidate the mechanism behind the polyandry variation that was found, it does provide additional support for the idea that mating patterns can show potentially large within-species variation. This result suggests that the next stage in studying these processes should focus on interactions between selection pressures associated with sexually antagonistic coevolution and life-history theory.
